# The Potential Mechanism of Curcumin in Treating Oral Squamous Cell Carcinoma Based on Integrated Bioinformatic Analysis

**DOI:** 10.1155/2023/8860321

**Published:** 2023-10-14

**Authors:** Wu Siyuan, Lv Xiaozhi, Wu Jialin, Haigang Wei, Shiwei Liu, Chen Zou, Jing Song, Li Xia, Ai Yilong

**Affiliations:** ^1^Foshan Stomatological Hospital, School of Medicine, Foshan University, Foshan, Guangdong, China; ^2^Department of Oral and Maxillofacial Surgery, Zhujiang Hospital, Southern Medical University, Guangzhou, China; ^3^Department of Stomatology, Foshan First People's Hospital, Foshan, Guangdong, China

## Abstract

**Aims:**

This study explores the effects of curcumin as a therapeutic agent against oral squamous cell carcinoma (OSCC).

**Methods:**

We acquired the targets of curcumin from three digital databases, including the Comparative Toxicogenomics Database, Search Tool for Interactions of Chemicals, and SwissTargetPrediction. Then, we identified the differentially expressed genes (DEGs) and the weighted gene coexpression network analysis-based key modules using the expression profiles of GSE23558 to acquire the OSCC-related genes. Additionally, the GeneCards and Online Mendelian Inheritance in Man databases were also used to identify the OSCC-related genes. Finally, curcumin-OSCC interaction genes were obtained by overlapping curcumin targets and OSCC-related genes. The enrichment analysis was performed by the ClusterProfiler algorithm and Metascape, respectively. Then, a protein-protein interaction network was created, and the maximal clique centrality algorithm was used to identify the top 10 hub genes. Besides, we examined the expression levels of hub genes in OSCC using The Cancer Genome Atlas database.

**Results:**

927 DEGs were identified, including 308 upregulated ones and 619 downregulated ones. The cluster one-step network construction function of the WGCNA algorithm recognized a soft-thresholding power of 6, and 9083 genes were acquired. 2591 OSCC-related genes were obtained by overlapping the GSE23558-identified genes and the OSCC-related genes from disease target bases. Finally, we identified 70 candidate drug-disease interaction genes by overlapping the disease-related genes with the curcumin target. The enrichment analysis suggested that response to oxidative stress, epithelial cell proliferation, and AGE/RAGE pathway might involve in the effect of curcumin on OSCC. The topologic study identified the ten hub genes, including *VEGFA*, *AKT1*, *TNF*, *HIF1A*, *EGFR*, *JUN*, *STAT3*, *MMP9*, *EGF*, and *MAPK3*. A significant difference was observed in VEGFA, AKT1, TNF, HIF1A, EGFR, MMP9, EGF, and MAPK3 expression levels between head and neck squamous cell carcinoma and the normal controls. However, no significant difference was observed in *JUN* (*P* = 0.14) and *STAT3* (*P* = 0.054).

**Conclusion:**

This study provided an overview and basis for the potential mechanism of curcumin against OSCC. The following experiments should be performed to further understand the effectiveness and safety of curcumin in treating OSCC.

## 1. Introduction

Oral squamous cell carcinoma (OSCC) is a malignant tumor originating from the oral cavity epithelium, accounting for over 90% of oral malignancies [1]. The primary sites of OSCC include the buccal mucosa, tongue, oral floor, and oropharynx. Dominant etiological contributors of OSCC encompass tobacco/alcohol consumption, human papillomavirus infection, and genetic predisposition. Currently, the therapies against OSCC include surgical intervention, radiotherapy, and chemotherapy [2]. Targeted therapy and immunotherapy have also been used as promising and practical approaches to augment treatment efficacy and ameliorate patient outcomes [3, 4]. Despite the ongoing effort on innovative treatment targets, prognostic indicators, and personalized therapy, it remains challenging to address the complexities of OSCC management and improve survival. Also, the therapeutic response is heterogeneous and is influenced by multiple factors such as tumor biology, genetic aberrations, and host immune response. Currently, patients with OSCC show a 5-year overall survival rate of below 50%, and OSCC results in over half a million deaths worldwide yearly [5, 6].

Curcumin is a polyphenolic compound derived from the rhizomes of Curcuma longa, which serves as the primary bioactive constituent of turmeric [7]. Curcumin has been extensively used in traditional Chinese medicine to treat various conditions, such as inflammation, pain, wounds, and gastrointestinal disorders [8]. Contemporary research has unveiled many pharmacological properties of curcumin, encompassing antioxidant, anti-inflammatory, antimicrobial, and anticancer activities [9]. Importantly, the potential anticancer effect of curcumin has been explored in diverse cancer types, including breast, colon, lung, prostate, and OSCC [10]. Despite the accumulating research, the potential targets and mechanisms of curcumin in OSCC treatment remain largely unclear.

Network pharmacology is a burgeoning interdisciplinary domain that converges system biology, network analysis, and computational biology with pharmacology [11, 12]. Recently, network pharmacology has been widely used to analyze the complex effects of herbal compounds and facilitate drug discovery [11]. Compared with traditional pharmacology, network pharmacology provides a network target and multicomponent therapeutic perspective, which enables the systematic investigation of interactions between bioactive constituents and their corresponding molecular targets within biological systems, thus facilitating the elucidation of synergistic effects and underlying mechanisms.

This study is aimed at exploring the effects of curcumin as a therapeutic agent against oral squamous cell carcinoma based on network pharmacology.

## 2. Materials and Methods

### 2.1. Identification of the Targets of Curcumin

**Figure 1:**
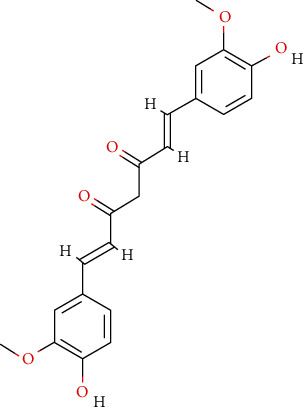
The 2D structure of curcumin. The canonical SMILES of curcumin is COC1=C(C=CC(=C1)C=CC(=O)CC(=O)C=CC2=CC(=C(C=C2)O)OC)O. PubChem CID: 969516.

We searched in the PubChem database using the keyword “Curcumin.” The PubChem CID is 969516, and the 2D structure (Figure 1) and canonical SMILES of curcumin were obtained.

Three databases were applied to identify the potential targets of curcumin, including Comparative Toxicogenomics Database (CTD) [13], Search Tool for Interactions of Chemicals (STITCH) [14], and SwissTargetPrediction [15]. All three databases have been widely used to explore pharmacological networks and perform drug discovery. The CTD is a publicly available resource recording the molecular mechanisms underlying environmental exposures and diseases, which provides information on interactions between chemicals, genes, and diseases [13]. STITCH is a comprehensive database integrating known and predicted interactions between proteins and small molecules [14]. STITCH incorporates data from multiple sources (chemical-protein binding, metabolic pathways, and text mining). It allows researchers to explore potential drug targets and their interactions in a user-friendly, visually rich interface [14]. SwissTargetPrediction is an online tool predicting potential protein targets of small molecules based on their chemical similarity to known ligands [15]. The compound name (curcumin) or the canonical SMILES of curcumin were input into the three databases, and the targets were acquired accordingly. It should be noticed that only genes with an interaction count above or equal to 10 were obtained from the CTD. The targets identified by any of the three databases were considered candidate curcumin targets.

### 2.2. Acquisition of OSCC-Related Genes

#### 2.2.1. Acquisition Strategy Based on Gene Expression Omnibus Database

Gene Expression Omnibus (GEO) is a public repository storing high-throughput gene expression and other functional genomic datasets, which provides a searchable platform to access, analyze, and visualize gene expression data. We acquired the gene expression profiling from 27 patients with oral squamous cell carcinoma and five control samples (series GSE23558, https://www.ncbi.nlm.nih.gov/geo/query/acc.cgi?acc=GSE23558). Neoprimary oral tumor samples were collected from patients undergoing surgery, and none received radiation or chemotherapy before surgery. The expression profiles included 27 patients with gingivobuccal complex cancer and five normal gingivobuccal complex samples (4 from normal individuals and one pool sample from 9 other normal samples). The platform of GSE23558 is GPL6480 (Agilent-014850 Whole Human Genome Microarray 4X44K G4112F). The study design has been previously provided [16].

Data preprocessing, including background correction and quantile normalization, was performed to ensure reliable downstream analysis using the linear models for microarray data (limma) package. Subsequently, the limma algorithm was employed to identify differentially expressed genes (DEGs) between the OSCC and the control group. The threshold value for significant DEGs was set as adjusted *P* < 0.05 and |log_2_(fold change)| > 2. The DEGs were illustrated by the heatmap and the volcano plot.

Weighted gene coexpression network analysis (WGCNA) is a bioinformatic algorithm that constructs gene networks based on gene expression and recognizes correlated gene modules. By focusing on coexpression patterns instead of individual genes, WGCNA reveals functional relationships and gene interactions underlying complex biological processes. WGCNA has been widely used to explore candidate biomarkers, therapeutic targets, and key regulatory genes. In our study, we performed the WGCNA to investigate gene interactions and functional relationships based on the top 25% of most variant genes of GSE23558. We first evaluated the presence of obvious outliers using cluster analysis. Next, we employed the one-step network construction function to create a coexpression network and identify key modules. Genes were hierarchically clustered and visualized in a dendrogram, with branches of the tree labeled with specific colors representing individual modules containing highly correlated genes. The gray section denoted background genes not belonging to any modules. To assess the significance of each module, we summarized the module eigengene (ME) based on the first principal component of the module expression. Module-trait relationships were determined by correlating MEs with clinical traits. We evaluated the correlation strength using module significance (MS), defined as the average absolute gene significance (GS) of all genes within a module. The GS value was calculated based on the log_10_ transformation of the *P* value in the linear regression between gene expression and clinical traits. Modules significantly associated with OSCC were considered key modules, which potentially harbored critical genes related to the development of OSCC.

#### 2.2.2. Acquisition Strategy Based on Disease Target Databases

Two online disease target databases were applied to identify OSCC-related genes, including GeneCards [17] and Online Mendelian Inheritance in Man (OMIM) [18], which have been widely used to facilitate the understanding of inheritance patterns and molecular mechanisms underlying diseases. GeneCards [17] is an integrative database on human gene-centric information, including gene function, expression patterns, and disease involvement, which supports the research on gene-disease associations and therapeutic targets. OMIM is a comprehensive, authoritative database focused on human genes and genetic disorders, which contains expert-curated information on phenotype-genotype relationships [18]. We searched in the GeneCards using “oral squamous cell carcinoma” as the keyword and in the OMIM using “head and neck squamous cell carcinoma.” We overlapped the DEGs, key modules, and database-related genes, and importantly, the overlapped genes were considered OSCC-related genes.

Finally, the genes in both the OSCC-related gene list and curcumin targets were identified as curcumin-OSCC interaction genes.

### 2.3. Enrichment Analysis

ClusterProfiler algorithm is developed for comprehensive functional annotation and enrichment analysis of gene clusters, which facilitates to analyze and visualize functional profiles of genomic coordinates, gene clusters, and gene expression data [19]. The identified drug-disease interaction genes were mapped to their corresponding Entrez IDs and subjected to Gene Ontology (GO) enrichment analysis by the ClusterProfiler package. Then, the enrichment analysis was conducted for each of the three GO categories: biological process (BP), molecular function (MF), and cellular component (CC). The *P* value was adjusted for multiple testing by the Benjamini-Hochberg procedure, and only GO terms with an adjusted *P* < 0.05 were considered significant. The top 10 most significant GO terms within each category were visualized.

Moreover, we performed a comprehensive functional enrichment analysis of the hub genes by the Metascape web-based tool [20]. Metascape integrates multiple databases, including Gene Ontology (GO), KEGG, UniProt, and Reactome, thus providing an in-depth functional annotation of gene clusters.

### 2.4. Construction of the Protein-Protein Interaction Network

The STRING (Search Tool for the Retrieval of Interacting Genes/Proteins) database is a comprehensive resource that provides information on known and predicted protein-protein interactions (PPIs), which integrates data from experimental repositories, computational predictions, and text mining of scientific literature [21]. We input the identified curcumin-OSCC interaction genes into the STRING using the “Multiple proteins” search option, and the organism was set as Homo sapiens. The minimum required interaction threshold of 0.7 was applied, indicating a high-confidence interaction. The resulting PPI network was visualized using the built-in tools provided by the STRING web interface.

CytoHubba is a widely used plugin for Cytoscape that provides a user-friendly interface to identify and rank hub genes within a network based on various topological algorithms [22]. We input the PPI network into the Cytoscape software, and then, we applied CytoHubba to perform the maximal clique centrality algorithm. The high topological scores indicate a high level of connectivity and potential biological significance within the network. The top 10 genes with the highest topological scores were identified as hub genes.

### 2.5. Clinical Significance of the Hub Genes

UALCAN is a comprehensive tool developed for the in-depth analysis of gene expression data from The Cancer Genome Atlas (TCGA) database, which provides an approach to analyzing gene expression data from TCGA and facilitating the identification of potential biomarkers, therapeutic targets, and insights into the molecular mechanisms of cancer [23]. We applied the UALCAN to evaluate the expression levels of the identified hub genes in head and neck squamous cell carcinoma and compare them with the normal control samples. Moreover, we conducted survival analyses according to the expression levels of the hub genes, which are aimed at evaluating the prognostic significance of the hub genes. The log-rank test was used to determine the statistical significance of the survival differences between the two groups.

## 3. Results

### 3.1. Candidate Targets of Curcumin

After compound-target database searching, we identified 81 targets from CTD, 10 from the STITCH, and 64 from the SwissTargetPrediction. After removing duplicate targets, 137 candidate curcumin targets were identified.

### 3.2. Candidate OSCC-Related Genes

**Figure 2:**
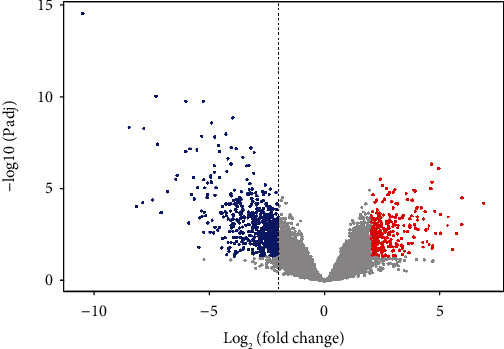
The volcano plot of all differentially expressed genes. The red dots indicate the upregulated genes, and the blue dots indicate the downregulated genes.

**Figure 3:**
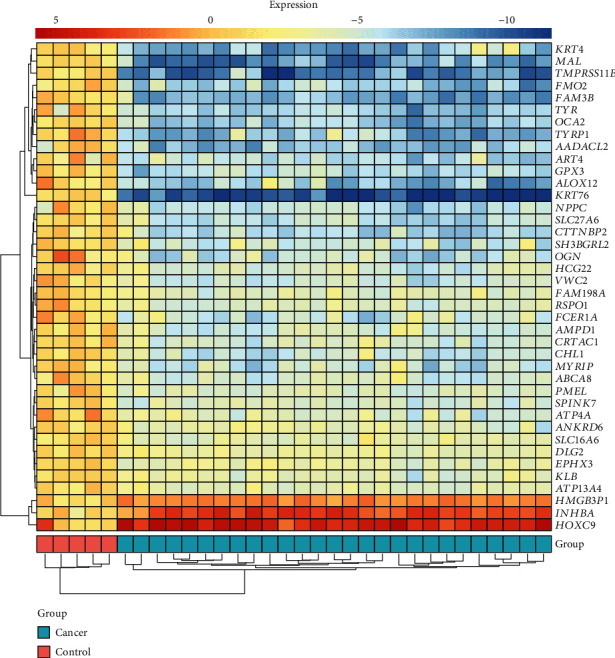
The heatmap of the top 40 differentially expressed genes.

We identified 927 DEGs, including 308 upregulated ones and 619 downregulated ones. The volcano plot of all DEGs is given in Figure 2, and the expression heatmap of the top 40 DEGs is shown in Figure 3.

**Figure 4:**
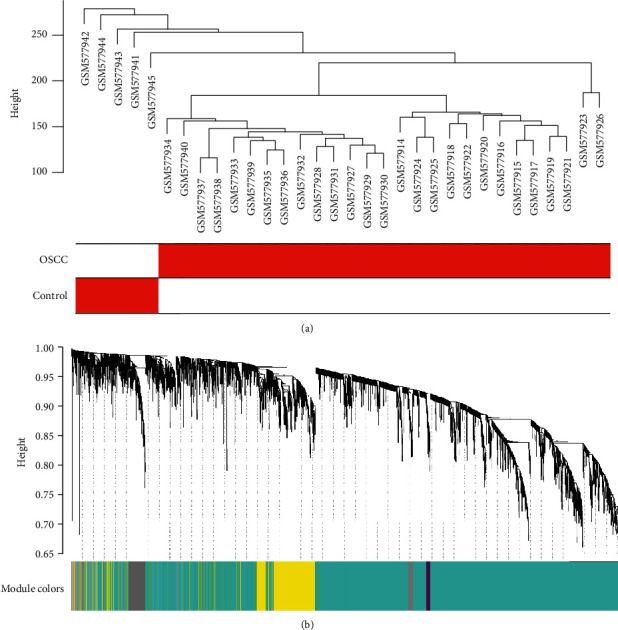
Sample clustering and network construction. (a) Clustering dendrogram of oral squamous cell carcinoma and control samples. (b) Dendrogram clustered based on a dissimilarity measure.

Using a scale-free network and topological overlaps, we constructed a hierarchical clustering tree based on the dynamic hybrid cut method (Figure 4(a)). Based on the scale-free topology criterion, the one-step network construction WGCNA algorithm identified a soft-thresholding power of 6 (scale-free *R*^2^ = 0.90). A total of 35 modules were created, and the dendrogram clustered by dissimilarity measure is shown in Figure 4(b).

**Figure 5:**
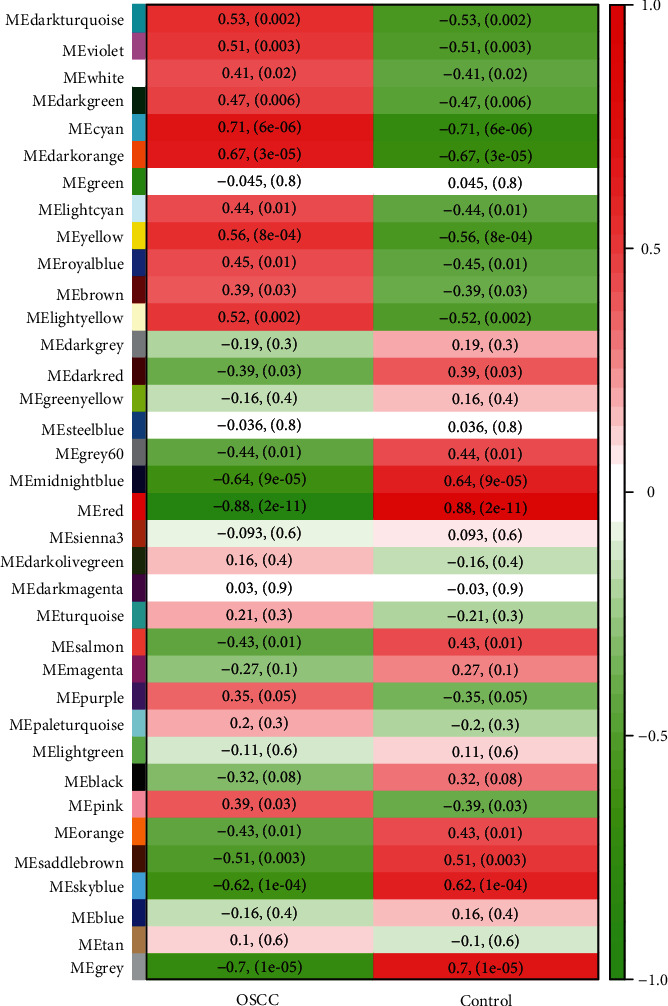
Heatmap of the correlation between module eigengenes and the OSCC.

Next, we analyzed the association between gene modules and OSCC. As shown in Figure 5, most modules were significantly associated with OSCC, but not the green, dark gray, green yellow, steel blue, sienna3, dark olive green, dark magenta, turquoise, magenta, pale turquoise, light green, blue, and tan modules. The modules with the significant association were identified as key modules, which included 9083 genes.

After removing duplicate targets, 9179 candidate disease-related genes were identified based on GSE23558.

Based on disease-related database searching, we identified 5530 targets from GeneCards and 163 from the OMIM database. After removing duplicate targets, a total of 5575 candidate disease-related genes were identified using database searching.

### 3.3. The Drug-Disease Interaction Genes

**Figure 6:**
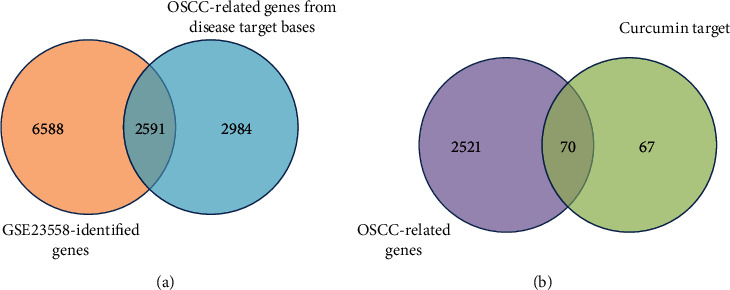
The Venn plot for curcumin-OSCC interaction genes. (a) A total of 2591 disease-related genes were acquired by overlapping the GSE23558-identified genes and the OSCC-related genes from disease target bases. (b) 70 candidate curcumin-OSCC interaction genes were obtained by overlapping the disease-related genes with the curcumin target. It should be noticed that duplicated genes have been removed from the same circle.

As shown in Figure 6, we first overlapped the GSE23558-identified genes (including DEGs and genes from the key modules) and the OSCC-related genes from disease target bases. A total of 2591 OSCC-related genes were acquired. Then, we overlapped the disease-related genes with the curcumin target, and 70 candidate curcumin-OSCC interaction genes were obtained.

### 3.4. Enrichment Analysis

**Figure 7:**
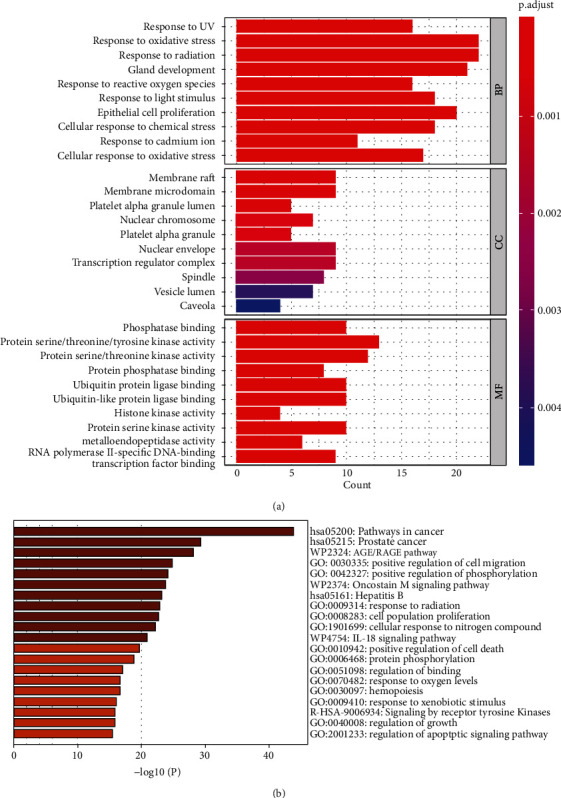
The enrichment analysis. (a) Gene Ontology enrichment analysis based on the candidate drug-interaction genes. The enrichment analysis was conducted for the three GO categories: biological process, molecular function, and cellular component. (b) The top 20 enriched terms based on Metascape.

The enrichment analysis according to the three GO categories is displayed in Figure 7(a). In the BP category, the top enriched GO terms were primarily related to response to UV, response to oxidative stress, response to radiation, gland development, response to reactive oxygen species, response to light stimulus, epithelial cell proliferation, and cellular response to chemical stress. In the CC category, the enrichment analysis identified key molecular functions such as membrane raft, membrane microdomain, platelet alpha granule lumen, nuclear chromosome, platelet alpha granule, nuclear envelope, transcription regulator complex, spindle, vesicle lumen, and caveola. The enriched MF terms included phosphatase binding, protein serine/threonine/tyrosine kinase activity, protein serine/threonine kinase activity, protein phosphatase binding, ubiquitin protein ligase binding, ubiquitin-like protein ligase binding, histone kinase activity, protein serine kinase activity, metalloendopeptidase activity, and RNA polymerase II-specific DNA-binding transcription factor binding.

Moreover, the top 20 enriched terms based on Metascape are shown in Figure 7(b). The enriched terms included pathways in cancer (log_10_(*q*) = −39.54), prostate cancer (log_10_(*q*) = −25.55), AGE/RAGE pathway (log_10_(*q*) = −24.41), positive regulation of cell migration (log_10_(*q*) = −21.64), positive regulation of phosphorylation (log_10_(*q*) = −21), oncostatin M signaling pathway (log_10_(*q*) = −20.65), hepatitis B (log_10_(*q*) = −20.11), response to radiation (log_10_(*q*) = −19.84), cell population proliferation (log_10_(*q*) = −19.69), cellular response to nitrogen compound (log_10_(*q*) = −19.22), IL-18 signaling pathway (log_10_(*q*) = −18.07), positive regulation of cell death (log_10_(*q*) = −17.05), protein phosphorylation (log_10_(*q*) = −16.2), regulation of binding (log_10_(*q*) = −14.59), response to oxygen levels (log_10_(*q*) = −14.19), hemopoiesis (log_10_(*q*) = −14.18), response to xenobiotic stimulus (log_10_(*q*) = −13.66), signaling by receptor tyrosine kinases (log_10_(*q*) = −13.48), regulation of growth (log_10_(*q*) = −13.46), and regulation of apoptotic signaling pathway (log_10_(*q*) = −13.1).

### 3.5. Protein-Protein Interaction Network

**Figure 8:**
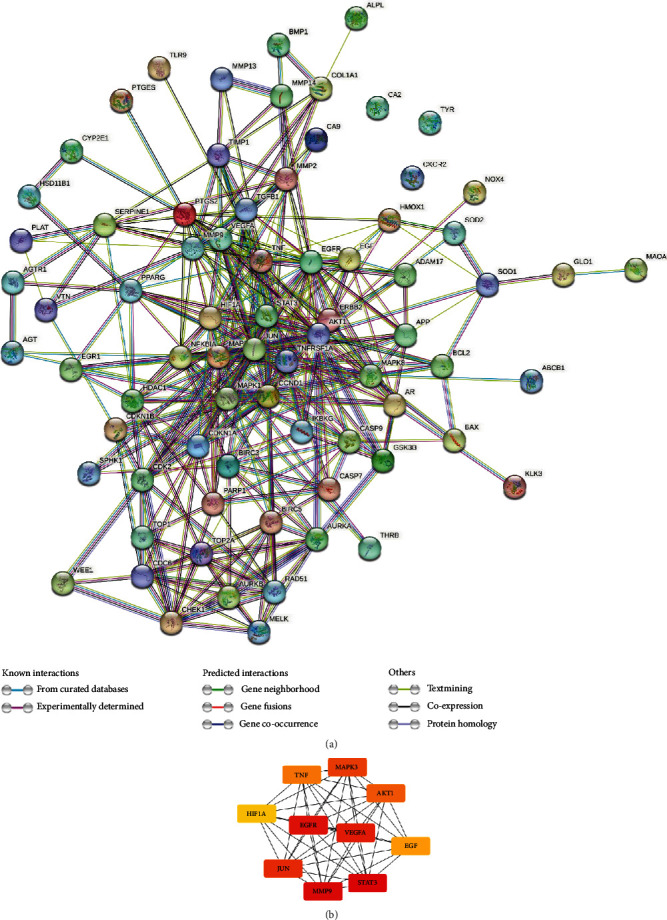
Protein-protein interaction network. (a) The protein-protein interaction network of the candidate curcumin-OSCC interaction genes. The network nodes represent proteins, while the edges represent interactions between the proteins. Different colors of the edges indicate various types of evidence supporting the interactions. (b) The top 10 hub genes identified by the maximal clique centrality algorithm include *VEGFA*, *AKT1*, *TNF*, *HIF1A*, *EGFR*, *JUN*, *STAT3*, *MMP9*, *EGF*, and *MAPK3*.

The created PPI network is displayed in Figure 8(a). The number of edges was 344, with an average node degree of 9.17. The hub genes identified by the maximal clique centrality algorithm were *VEGFA*, *AKT1*, *TNF*, *HIF1A*, *EGFR*, *JUN*, *STAT3*, *MMP9*, *EGF*, and *MAPK3* (Figure 8(b)). The introduction to the hub genes is provided in Supplement Table 1.

### 3.6. Clinical Significance of the Hub Genes

**Figure 9:**
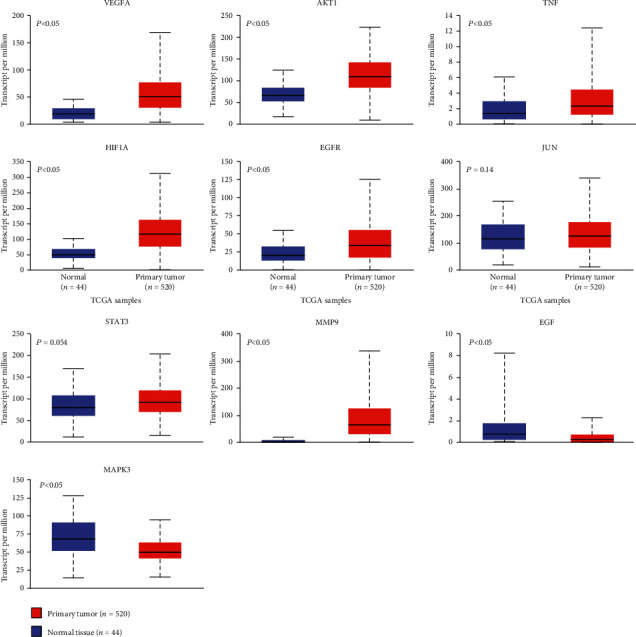
The expression levels of the ten hub genes in the head and neck squamous cell carcinoma compared with normal control samples.

The expression levels of the ten hub genes are shown in Figure 9. VEGFA, AKT1, TNF, HIF1A, EGFR, MMP9, EGF, and MAPK3 expression levels in head and neck squamous cell carcinoma significantly differed from the normal controls (all *P* < 0.05). However, no significant difference was observed in *JUN* (*P* = 0.14) and *STAT3* (*P* = 0.054). The survival analysis showed no significant survival difference in patients with low or high expression levels of hub genes (all *P* > 0.05).

## 4. Discussion

Curcumin, the principal constituent of turmeric, has exhibited potential antitumor properties in clinical trials targeting malignancies such as hepatic, colorectal, and mammary neoplasms [24]. In this study, we used a network pharmacology strategy to explore the potential mechanisms underlying the effect of curcumin on OSCC.

The GO enrichment showed that the anticancer effect of curcumin on OSCC might act in membrane raft, membrane microdomain, platelet alpha granule lumen, nuclear chromosome, etc. The molecular functions involved were phosphatase binding, protein serine/threonine/tyrosine kinase activity, and protein serine/threonine kinase activity. Additionally, curcumin exerts its effect against OSCC by affecting biological processes, including response to oxidative stress, response to radiation, gland development, response to reactive oxygen species, response to light stimulus, epithelial cell proliferation, and cellular response to chemical stress. Moreover, the enriched terms by Metascape included the AGE/RAGE pathway, positive regulation of cell migration, positive regulation of phosphorylation, cell population proliferation, cellular response to nitrogen compound, IL-18 signaling pathway, positive regulation of cell death, protein phosphorylation, and regulation of apoptotic signaling pathway.

The GO BP analysis enriched the term of cell proliferation. Cell proliferation is the process by which cells grow and divide, increasing the number of cells, which plays a vital role in organism growth and tissue repair. However, the disturbed proliferation of squamous epithelial cells lining the oral cavity can result in the development of OSCC. Liu et al. [25] investigated the effect of curcumin nanoemulsions on cell proliferation using OSCC HSC-3 cell lines. The dose- or time-dependent decrease was observed in the percentage of cells in proliferative phases. Additionally, curcumin nanoemulsions inhibited the cell proliferation induced by miR-199a inhibitor [25]. In another *in vitro* study on HSC3 and CAL33 cells, the treatment with curcumin significantly inhibited the viability and colony formation ability of OSCC cells, and the expression of specificity protein 1, p65, and heat shock factor 1 was substantially reduced [26]. Similarly, treating HO-3867 (a novel curcumin analog) on phase human OSCC SCC-9 and HSC-3 cells could suppress the cell proliferation and induce the sub-G1 [27].

Apoptosis, also known as programmed cell death, is a crucial process of eliminating damaged, aged, or unnecessary cells from the body to maintain tissue homeostasis [28]. The dysregulation of apoptosis could contribute to evading apoptosis, tumor growth, progression, and resistance to the treatment of OSCC. Chen et al. reported that curcumin analog could induce OSCC cell apoptosis via activating caspase 3, caspase 8, caspase 9, and PARP [27]. Similarly, diphenyl difluoroketone (a curcumin analog) could downregulate the cell viability and increase the levels of activated caspase 3 and caspase 9 [29].

The topologic analysis identified the ten hub genes that might be the target of curcumin against OSCC. The hub genes included *VEGFA*, *AKT1*, *TNF*, *HIF1A*, *EGFR*, *JUN*, *STAT3*, *MMP9*, *EGF*, and *MAPK3*. Hypoxia is a common feature of the cancer microenvironment and contributes to OSCC development, progression, and therapy resistance [30], where hypoxia-inducible factor 1 (HIF-1) is a key transcription factor that regulates cellular response to hypoxia [31]. Under hypoxic conditions, the activated HIF-1 transcriptional complex would upregulate the transcription and expression of vascular endothelial growth factor A (VEGFA) [32]. Subsequently, VEGFA exerts its proangiogenic effects and activates the stimulation of endothelial cell proliferation, migration, and survival [33]. Our topologic research identified the *HIF1A* and *VEGFA* as potential therapeutic targets of curcumin against OSCC.

Despite the anticancer effect of curcumin, its bioavailability is hindered by its physicochemical characteristics, such as high lipophilicity and photosensitivity [34]. The poor absorption, quick metabolism, and fast systemic elimination result in low plasma and tissue levels after curcumin treatment. To overcome the limitation in the bioavailability of curcumin, continuous efforts are being made in this area. Recently, an emulgel system was developed to deliver curcumin, which was prepared with poloxamer 407, acrylic acid derivatives, and oil phase [35]. The emulgel system offers several advantages for the delivery of curcumin in treating oral cancer, including sustained contact with the oral mucosa, pseudoplastic behavior and gelation temperature, controlled drug release, and enhanced permeation.

The limitations of this study should be noticed. Given the growing number of accessible gene expression profiles related to diseases, there is a pressing need to effectively and economically harness these vast resources for thorough analysis. Nonetheless, delving deeper into mechanisms based solely on our current dataset may not yield reliable insights. As such, our study remains primarily observational and lacks detailed mechanistic exploration. To corroborate our findings, subsequent molecular biology experiments are still necessary.

## 5. Conclusion

This *in silico* study provided an overview and basis for the potential mechanism of curcumin against OSCC. Further biological experiments should be performed to fully understand its effectiveness and safety in treating OSCC before the clinical trials.

## Data Availability

The data used to support the findings of this study are available from the corresponding author upon request. The data can also be available from https://www.ncbi.nlm.nih.gov/geo/.

## Supplementary Materials

Supplementary Materials Supplement Table 1: the introduction to the hub genes based on GeneCards.
