# Supplementary material for: The Potential Mechanism of Curcumin in Treating Oral Squamous Cell Carcinoma Based on Integrated Bioinformatic Analysis
Source: Int J Genomics. 2023 Oct 14;2023:8860321. doi: 10.1155/2023/8860321 (PMC10590272; doi:10.1155/2023/8860321)
Supplement: Supplementary Materials — Supplement Table 1: the introduction to the hub genes based on GeneCards. [file 8860321.f1.docx]

**Supplement Table 1**. The introduction to the hub genes based on GeneCards.

| Gene symbol | GeneCards Summary |
| --- | --- |
| *VEGFA* | VEGFA (Vascular Endothelial Growth Factor A) is a Protein Coding gene. Diseases associated with VEGFA include Microvascular Complications Of Diabetes 1 and Poems Syndrome. Among its related pathways are Response to elevated platelet cytosolic Ca2+ and VEGF Pathway (Qiagen). Gene Ontology (GO) annotations related to this gene include protein homodimerization activity and protein heterodimerization activity. An important paralog of this gene is PGF. |
| *AKT1* | AKT1 (AKT Serine/Threonine Kinase 1) is a Protein Coding gene. Diseases associated with AKT1 include Proteus Syndrome and Cowden Syndrome 6. Among its related pathways are Prolactin Signaling and Regulation of activated PAK-2p34 by proteasome mediated degradation. Gene Ontology (GO) annotations related to this gene include identical protein binding and protein kinase activity. An important paralog of this gene is AKT3. |
| *TNF* | TNF (Tumor Necrosis Factor) is a Protein Coding gene. Diseases associated with TNF include Malaria and Asthma. Among its related pathways are MIF Mediated Glucocorticoid Regulation and TNFR1 Pathway. Gene Ontology (GO) annotations related to this gene include identical protein binding and cytokine activity. An important paralog of this gene is TNFSF15. |
| *HIF1A* | HIF1A (Hypoxia Inducible Factor 1 Subunit Alpha) is a Protein Coding gene. Diseases associated with HIF1A include Retinal Ischemia and Enchondromatosis, Multiple, Ollier Type. Among its related pathways are Signaling by PTK6 and Regulation of activated PAK-2p34 by proteasome mediated degradation. Gene Ontology (GO) annotations related to this gene include DNA-binding transcription factor activity and protein heterodimerization activity. An important paralog of this gene is EPAS1. |
| *EGFR* | EGFR (Epidermal Growth Factor Receptor) is a Protein Coding gene. Diseases associated with EGFR include Inflammatory Skin And Bowel Disease, Neonatal, 2 and Lung Cancer. Among its related pathways are Apoptotic Pathways in Synovial Fibroblasts and Prolactin Signaling. Gene Ontology (GO) annotations related to this gene include identical protein binding and protein kinase activity. An important paralog of this gene is ERBB4. |
| JUN | JUN (Jun Proto-Oncogene, AP-1 Transcription Factor Subunit) is a Protein Coding gene. Diseases associated with JUN include Breast Cancer and Sarcoma. Among its related pathways are MyD88 dependent cascade initiated on endosome and Prolactin Signaling. Gene Ontology (GO) annotations related to this gene include RNA binding and sequence-specific DNA binding. An important paralog of this gene is JUND. |
| STAT3 | STAT3 (Signal Transducer And Activator Of Transcription 3) is a Protein Coding gene. Diseases associated with STAT3 include Hyper-Ige Recurrent Infection Syndrome 1, Autosomal Dominant and Autoimmune Disease, Multisystem, Infantile-Onset, 1. Among its related pathways are IL-9 Signaling Pathways and Prolactin Signaling. Gene Ontology (GO) annotations related to this gene include DNA-binding transcription factor activity and sequence-specific DNA binding. An important paralog of this gene is STAT1. |
| MMP9 | MMP9 (Matrix Metallopeptidase 9) is a Protein Coding gene. Diseases associated with MMP9 include Metaphyseal Anadysplasia 2 and Metaphyseal Anadysplasia. Among its related pathways are Collagen chain trimerization and Matrix metalloproteinases. Gene Ontology (GO) annotations related to this gene include identical protein binding and metalloendopeptidase activity. An important paralog of this gene is MMP2. |
| *EGF* | EGF (Epidermal Growth Factor) is a Protein Coding gene. Diseases associated with EGF include Hypomagnesemia 4, Renal and Egf-Related Primary Hypomagnesemia With Intellectual Disability. Among its related pathways are Apoptotic Pathways in Synovial Fibroblasts and Signaling by EGFR in Cancer. Gene Ontology (GO) annotations related to this gene include calcium ion binding and epidermal growth factor receptor binding. An important paralog of this gene is LRP1B. |
| *MAPK3* | MAPK3 (Mitogen-Activated Protein Kinase 3) is a Protein Coding gene. Diseases associated with MAPK3 include B-Cell Lymphoma and Malignant Pleural Mesothelioma. Among its related pathways are Prolactin Signaling and MyD88 dependent cascade initiated on endosome. Gene Ontology (GO) annotations related to this gene include transferase activity, transferring phosphorus-containing groups and protein tyrosine kinase activity. An important paralog of this gene is MAPK1. |
